# Fine-Tuning the Nanostructured Titanium Oxide Surface for Selective Biological Response

**DOI:** 10.1021/acsabm.3c00686

**Published:** 2023-12-08

**Authors:** Niharika Rawat, Metka Benčina, Domen Paul, Janez Kovač, Katja Lakota, Polona Žigon, Veronika Kralj-Iglič, Hsin-Chia Ho, Marija Vukomanović, Aleš Iglič, Ita Junkar

**Affiliations:** † Laboratory of Physics, Faculty of Electrical Engineering, University of Ljubljana, Tržaška 25, SI-1000 Ljubljana, Slovenia; ‡ Department of Surface Engineering, Jožef Stefan Institute, Jamova 39, SI-1000 Ljubljana, Slovenia; § Department of Rheumatology, University Medical Centre Ljubljana, Vodnikova 62, SI-1000 Ljubljana, Slovenia; ∥ Laboratory of Clinical Biophysics, Faculty of Health Sciences, University of Ljubljana, Zdravstvena pot 5, SI-1000 Ljubljana, Slovenia; ⊥ Advanced Materials Department, Jožef Stefan Institute, Jamova 39, SI-1000 Ljubljana, Slovenia; # Chair of Orthopaedic Surgery, Faculty of Medicine, University of Ljubljana, Vrazov trg 2, SI-1000 Ljubljana, Slovenia

**Keywords:** gaseous plasma treatment, hydrothermal treatment, biocompatibility, vascular stent, nanostructured surface

## Abstract

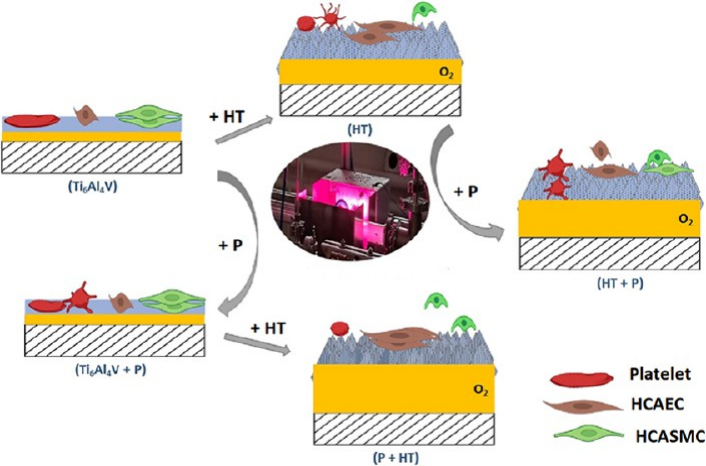

Cardiovascular diseases are a pre-eminent global cause of mortality in the modern world. Typically, surgical intervention with implantable medical devices such as cardiovascular stents is deployed to reinstate unobstructed blood flow. Unfortunately, existing stent materials frequently induce restenosis and thrombosis, necessitating the development of superior biomaterials. These biomaterials should inhibit platelet adhesion (mitigating stent-induced thrombosis) and smooth muscle cell proliferation (minimizing restenosis) while enhancing endothelial cell proliferation at the same time. To optimize the surface properties of Ti_6_Al_4_V medical implants, we investigated two surface treatment procedures: gaseous plasma treatment and hydrothermal treatment. We analyzed these modified surfaces through scanning electron microscopy (SEM), water contact angle analysis (WCA), X-ray photoelectron spectroscopy (XPS), and X-ray diffraction (XRD) analysis. Additionally, we assessed in vitro biological responses, including platelet adhesion and activation, as well as endothelial and smooth muscle cell proliferation. Herein, we report the influence of pre/post oxygen plasma treatment on titanium oxide layer formation via a hydrothermal technique. Our results indicate that alterations in the titanium oxide layer and surface nanotopography significantly influence cell interactions. This work offers promising insights into designing multifunctional biomaterial surfaces that selectively promote specific cell types’ proliferation—which is a crucial advancement in next-generation vascular implants.

## 1 Introduction

According to the United Nations report, the global population of 60 years and over is predicted to reach approximately 2.1 billion people by 2050.^1^ With an increase in global aging, a direct increment in the number of chronic diseases is also observed. For instance, bone and joint inflammatory and degenerative problems and cardiovascular diseases are present in more than half of all chronic diseases. Cardiovascular diseases (CVD) have nearly doubled in number from 271 million in 1990 to 523 million in 2019.^2^ Diverse treatment options have been explored for CVDs, ranging from administering drugs to surgical intervention that usually consists of tissue transplantation or bypass grafts. With the application and invention of coronary stents, a dramatic decrease in bypass surgeries was reported. Drug-eluting stents (DESs) were developed to replace conventionally used bare metal stents (BMSs),^3^ as they caused complications such as reblockage of vessels, called in-stent restenosis.^4,5^ After stent implantation, approximately 20–30% of patients experienced in-stent restenosis.^6^ This post-implant complication is due to vascular smooth cell proliferation, migration, and growth of the arterial inner wall because of the insufficient biocompatibility of stent material.^7^ Bioresorbable vascular stents or scaffolds (BRS) were developed to solve the problem of restenosis; however, only limited success was achieved and the prognosis for the future is that BMS and DES will remain the predominant vascular stents on the market.^8^

Blood-contacting medical devices (such as vascular stents) made of metals like nitinol, cobalt chromium, titanium, and alloys lack the desired biocompatibility due to thrombosis and restenosis. The adhesion of platelets is an indicator of surface hemocompatibility: the lower the platelet adhesion and their activation on the surface, the higher the material’s biocompatibility with blood.^9^ Hence, it is required that an ideal stent/implant material should primarily inhibit platelet adhesion and activation and also inhibit migration and proliferation of smooth muscle cells, and improve the integrity and viability of the endothelial cell layer.^10,11^ Several surface modification methods based on several types of coatings (organic and inorganic) have been recommended. These coatings alter the physicochemical properties of the surface such as roughness, morphology, surface chemistry, and wettability, which affect interaction with the biological environment.^12−14^ The surface modification via nanostructuring can be achieved using various methodologies, for instance, electrospinning,^15,16^ electrochemical anodization,^17−21^ sandblasting,^22,23^ nonthermal plasma treatment,^24^ and hydrothermal treatment.^10,25,26^ Titanium and its alloys are commonly used in cardiovascular applications such as stents and artificial heart valves due to their biocompatibility, lightweight, nonmagnetic, and excellent corrosion resistance^27,28^ properties. Among titanium alloys, Ti_6_Al_4_V alloy is the most commonly used, studied, economical, and widely available alloy. However, upon contact with blood, the surface of titanium alloy interacts with plasma proteins,^21,29^ platelets, and red blood cells, leading to thrombosis.^30−32^ The biocompatibility, antibacterial property, corrosion, and wear resistance of Ti_6_Al_4_V alloy can be improved by adjusting the surface modification method. Karpagavalli et al.^33^ described that nanotopography produced on Ti_6_Al_4_V via deposition of nanostructured TiO_2_ enhanced biocomptability by reducing the integration of human aortic smooth muscle cells. Lin et al.^34^ coated Ti_6_Al_4_V with Ta_2_O_5_-containing carbon nanotubes via the atmospheric plasma spraying technique. They found that adding this coating improves indentation fracture toughness without degrading the inherent biological property of the material. Zhang et al.^35^ applied a mechanical method called friction stir processing to prepare nanocomposite layers of TiO_2_ and Ti_6_Al_4_V, which showed improvement in biocompatibility, corrosion resistance, and surface microhardness in comparison to control samples. In another work, El Hadad et al.^36^ prepared hydroxyapaptite (Hap) coatings on Ti_6_Al_4_V surface via the sol–gel method. It was observed that the prepared powders were nanocrystalline HA and were slightly different from the one present in human bone. Llopis-Grimalt et al.^37^ compared two different approaches: electrochemical anodization and addition of a quercetin coating on titanium surface. It was observed that nanostructuring induces an increase in surface roughness, which in turn improves the biocompatibility of BMS on endothelial cells and reduces platelet adhesion. It has already been reported that an appropriate nanostructured surface could influence platelet adhesion. This is due to the altered conformation of adsorbed proteins on the surface, mainly fibrinogen,^38^ a major platelet activation and adhesion-determining factor. Underlying that other physicochemical properties of surface such as wettability and chemical composition also play a major role in platelet interaction. Therefore, plasma treatment can also be used for optimization of the nanostructured surface properties. This treatment is performed using various gaseous discharges to induce varied surface charge, chemical composition, crystallinity, and surface roughness.^39,40^ These factors play an important role in immobilizing cells and proteins.^41^ Studies show that the nanoscale surface morphology can be easily achieved on the surface of Ti_6_Al_4_V alloy through the hydrothermal method.^42−44^

The hydrothermal method is commonly used as it is scalable, cost-effective, and considerably easier to use.^10^ Hydrothermal treatment (HT) of titanium alloy enables the formation of nanotopography, which could reduce platelet aggregation and adhesion and lead to minimal hemolysis. Nowadays, gaseous plasma-based surface modification techniques have received much attention as they present a rapid, simple, eco-friendly, and substrate-independent approach for modification of metal biomaterials.^45,46^ Griesser et al.^47^ provide an excellent review of plasma treatment and cell adhesion. In our previous work, we prepared nanostructured surfaces through hydrothermal treatment of Ti and alloys, i.e., Ti foil, Ti_6_Al_4_V, and NiTi.^25^ We reported about the effect of surface properties (elemental composition, morphology, and wettability) of these nanostructured materials on the bacterial activity. All of the surfaces demonstrated antibacterial activity due to altered morphology; the nanofeatures on the surface of hydrothermally treated samples can mechanically rupture the bacterial cells. As the best results were achieved for Ti_6_Al_4_V+ HT, this prompted us to further study the effect of gaseous plasma treatment in combination with hydrothermal treatment on Ti_6_Al_4_V and to evaluate their biocompatibility.

In this work, we have envisaged a novel methodology that combines two eco-friendly approaches, i.e., hydrothermal and cold plasma method, to achieve the TiO_2_ nanostructure on Ti_6_Al_4_V. The combination of both methods could provide additional benefits, such as the additional nanostructuring and altered surface chemistry. The main aim of this study is to investigate the effects of altered Ti_6_Al_4_V surface nanotopography and chemical composition on the interactions with whole blood, endothelial, and smooth vascular cells. We have modified the titanium surface through a combination of hydrothermal treatment and treatment with reactive oxygen plasma. This technique enabled the formation of a nanostructured titanium oxide layer with enhanced oxygen concentration on the surface of a metallic substrate. The efficacy of the hydrothermal and plasma treatment of Ti_6_Al_4_V substrates was demonstrated by hemocompatibility studies and the evaluation of human coronary artery endothelial cell (HCEC) and human coronary artery smooth muscle cell (HCASMC) proliferation.

## 2 Experimental Section

### 2.1 Sample Preparation

Ti_6_Al_4_V disc (diameter 16 mm) was washed in acetone, ethanol, and water (10 min each) inside the beaker and subjected to ultrasound. Afterward, the sample was dried at 70 °C in the furnace on the crucible for 1 h.

### 2.2 Hydrothermal Method (HT)

An aqueous solution (30 mL) containing 2 mL of titanium(IV) isopropoxide was prepared using *d*·H_2_O and NaOH to adjust the pH of the solution to 12. Thereafter, the prepared aqueous solution of titanium isopropoxide was poured onto the cleaned and dried Ti_6_Al_4_V disc kept inside a Teflon vessel. The Teflon vessel was sealed inside a stainless steel reactor (Paar, Ashland, VA) and kept inside the furnace at the temperature of 200 °C for 24 h, which was then cooled to room temperature naturally. After the hydrothermal synthesis, the Ti_6_Al_4_V disc was washed with deionized H_2_O and then ultrasonicated for 5 min followed by drying in an oven in an air atmosphere at 70 °C for 2 h.

### 2.3 Plasma Treatment (P)

The Ti_6_Al_4_V disc was pre- or post-treated with plasma using the following plasma parameters (RF plasma system, glass tube, quartz holder placed in the middle of the RF coil, and base pressure 2 Pa). Low pressure, radio frequency (RF), and inductively coupled plasma were used for plasma treatment of the Ti_6_Al_4_V disc. RF plasma was ignited inside a glass reactor and used for treatment with oxygen and hydrogen as gas carriers. Plasma treatment was done in two stages, starting with 10 s of H-mode hydrogen plasma at the pressure of 27 Pa (base pressure 2 Pa) and power of 600 W and with the addition of oxygen in the second step, lasting for 20 s at a combined pressure of 30 Pa (2 Pa base pressure, up to 27 Pa hydrogen and 30 Pa oxygen).

### 2.4 Incubation of the Samples with Whole Blood

The adhesion and activation of platelets on the samples were done according to the following procedure. Tests were performed following the Declaration of Helsinki and approved by Slovenia’s Ethics Committee (approval number 56/03/10). Prior to whole blood incubation, samples were cleaned with ethanol, dried, and incubated with whole blood taken by vein puncture from a healthy human donor. The blood was drawn into 9 mL tubes coated with trisodium citrate anticoagulant. Afterward, the fresh blood (250 μL) was incubated with samples in 24-well plates for 45 min at room temperature. After incubation, 250 μL of phosphate-buffered saline (PBS) was added to the whole blood. The blood with PBS was then removed, and the titanium surface was rinsed 5 times with 250 μL of PBS in order to remove weakly adherent platelets. Adherent cells were subsequently fixed with 250 μL of a 0.5% GA (glutaraldehyde) solution for 15 min at room temperature. Afterward, the surfaces were rinsed with PBS and then dehydrated using a graded ethanol series (50, 70, 80, 90, 100, and again 100 vol % ethanol) for 5 min and in the last stage in the series (100 vol % ethanol) for 15 min. Then the samples were placed in a critical point dryer, where the solvent was exchanged with liquid carbon dioxide. On increasing the temperature in the dryer state, the liquid carbon dioxide passes the critical point, at which the density of the liquid equals the density of the vapor phase. This drying process preserves the natural structure of the sample and avoids the surface tension that could be caused by normal drying. The dried samples were subsequently coated with gold/palladium and examined by means of SEM. The test was performed in triplicate, and only representative images are shown in this paper.

### 2.5 Cell Culture

Human coronary artery endothelial cells (HCEC) and human coronary artery smooth muscle cells (HCASMC) were purchased from Lifeline Cell Technology (Frederick, MD, USA). HCEC and HCASMC were plated into 75 cm^2^ flasks (TPP, Trasadigen, Switzerland) at 37 °C in a humidified atmosphere at 5% CO_2_ and grown in a VascuLife EnGS endothelial medium complete kit (Frederick, MD) or VascuLife SMC medium (ProVitro AG, Berlin, Germany), respectively, following the manufacturer’s instructions. For experiments, subconfluent cell cultures were used between passages 3 and 6.

### 2.6 Characterization

#### 2.6.1 Scanning Electron Microscopy (SEM)

The morphological analysis of the materials was conducted by a scanning electron microscope (JEOL JSM-7600F) at an accelerating voltage of 5–15 kV. The test was done in triplicate and only representative images are shown.

#### 2.6.2 Water Contact Angle (WCA) Analysis

The surface wettability was performed with Drop Shape Analyzer DSA-100 (Krüss GmbH, Hannover, Germany) by a sessile drop method to measure the static contact angle. The contact angle on the surface was analyzed immediately after plasma treatment by adding a 2.5 μL drop of deionized water on 8 different areas of the surface. Three measurements were performed for each sample, and the average value was calculated. The relative humidity was around 45% and the operating temperature was 21 °C, which did not vary significantly during continuous measurements.

#### 2.6.3 X-ray Photoelectron Spectroscopy (XPS)

The X-ray photoelectron spectroscopy (XPS) analyses were carried out using a PHI-TFA XPS spectrometer produced by Physical Electronics Inc. Samples were put on the sample holder and introduced into the ultrahigh vacuum spectrometer. The analyzed area was 0.4 mm in diameter and the analyzed depth was about 3–5 nm. Sample surfaces were excited by X-ray radiation from a monochromatic Al source at a photon energy of 1486.6 eV. XPS depth profile analyses were performed to get the concentration curves of elements in the surface layer of about 20–30 nm thickness. Ar ion sputtering with an ion energy of 1 keV was applied. The sputtering rate was about 2 nm/min.

#### 2.6.4 Immunofluorescent Microscopy and Cell Morphology

The HCEC and HCASMC were seeded on round disks of sample materials in 12-well plates at a density of 20,000 cells/cm^–2^ and grown for 2 days. The test was conducted in biological duplicates. Staining with fluorescein phalloidin (Invitrogen) was performed following the manufacturer’s instructions. Briefly, cells were washed 2 times for 3 min with PBS, pH 7.4, fixed in 3.7% formaldehyde solution for 10 min, and washed 3 times for 3 min with PBS at room temperature. Cells were incubated in 0.1% Triton X-100 for 4 min and then washed with PBS 3 times for 3 min. The dye stock was diluted 1:67 in PBS with 1% BSA and applied to HCEC and HCASMC for 30 min. The final washing steps were performed 3 times for 3 min with PBS. ProLong Diamond Antifade Mountant with DAPI (Thermo Fisher Scientific) was applied to HCEC and HCASMC (1 drop) and covered with a coverslip. Slides were examined on the same day. Images were generated using the fluorescent microscope Nikon eclipse E400 and a digital camera (Nikon Instruments, Dusseldorf, Germany). Analysis was performed with Nikon ACT-1 imaging software; the representative images are presented. In addition, the number of HCEC and HCASMC in round and spread forms was evaluated from the images taken by immunofluorescent microscopy. For each sample, 3 images were analyzed and their number was averaged.

## 3 Results and Discussion

**Figure 1:**
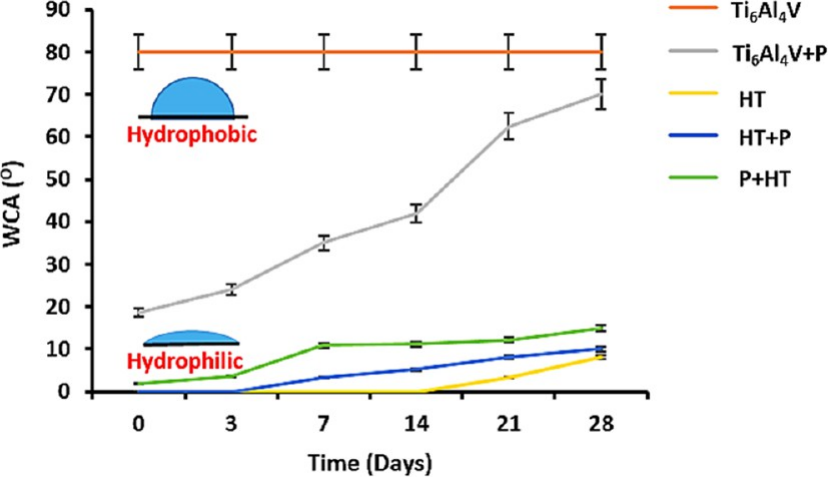
Aging study of the Ti_6_Al_4_V samples.

The results of WCA analysis of the untreated (Ti_6_Al_4_V), plasma-treated (Ti_6_Al_4_V + P), hydrothermally treated (HT), hydrothermally and plasma-treated (HT + P), and plasma-treated followed by hydrothermally treated (P + HT) Ti_6_Al_4_V are presented in Figure 1. The graph shows that the HT-, HT + P-, and P + HT-treated surfaces were all fully hydrophilic as the water drop nearly completely spread over the surface. For all freshly prepared treated samples, the surface was fully wettable (i.e., <5°) until 1 week of aging in air. After 3 weeks of aging, the samples were still hydrophilic; however, their contact angle slightly increased. For the HT sample, even after a month of aging, the contact angle was about 50°, while for HT + P and P + HT, the contact angle increased to about 8 and 12°, respectively. The most pronounced aging was observed for the plasma-treated surface (Ti_6_Al_4_V + P), where after 4 weeks of aging the contact angle increased to 60°. The untreated Ti_6_Al_4_V sample, however, was hydrophobic, with the contact angle of about 80°.

From X-ray photoelectron (XPS) analysis (Table 1), it can be determined that the surface of the untreated Ti_6_Al_4_V substrate is composed of about 44 atomic % oxygen, 11.7 atomic % titanium, and 40.7 atomic % carbon, while smaller amounts of N, Al, and V are also observed on the surface. Oxygen concentration was increased on all HT- and/or plasma-treated surfaces, while the most prominent increase was observed on the hydrothermally treated (HT) surface. Interestingly, oxygen concentrations on all HT surfaces, even on those pretreated with plasma (P + HT), were similar, about 55 atomic %. The highest Ti/O ratios were measured for the HT + P- and P-treated surfaces, 0.45 and 0.39, respectively. According to XPS analysis, plasma treatment increases the Al concentration, which was the highest in the case of Ti_6_Al_4_V + P, while similar values were found for the untreated and HT + P samples. This indicates that the final plasma treatment step, even in the case of the HT sample, increases the Al concentration on the surface. On the other hand, the plasma-pretreated followed by HT-treated (P + HT) sample exhibits almost negligible concentration of Al (0.6 atomic %) on the surface, similar to the HT sample. Increase of Na is observed after HT treatment, due to the use of sodium hydroxide for the HT treatment. After plasma treatment of the HT surface, a decrease in Na and an increase in Al are observed.

**Table 1:** Surface Chemical Composition in Atomic % Obtained by XPS Analysis of Ti_6_Al_4_V Samples

	atomic %								
material	C	O	Ti	N	Al	V	Na	Ti/O	Ti(3+)
Ti_6_Al_4_V	40.7	44.4	11.7	1.3	1.8	0.2	0	0.26	0
Ti_6_Al_4_V + P	21.0	52.8	20.5	1.0	3.5	0.1	1.1	0.39	8
HT	9.8	56.6	20.3	0	0	0	13.3	0.36	0
HT + P	10.6	56.2	25.1	0.2	1.2	0	6.8	0.45	9
P + HT	12.7	54.7	19.0	0	0.6	0	13.1	0.35	0

**Figure 2:**
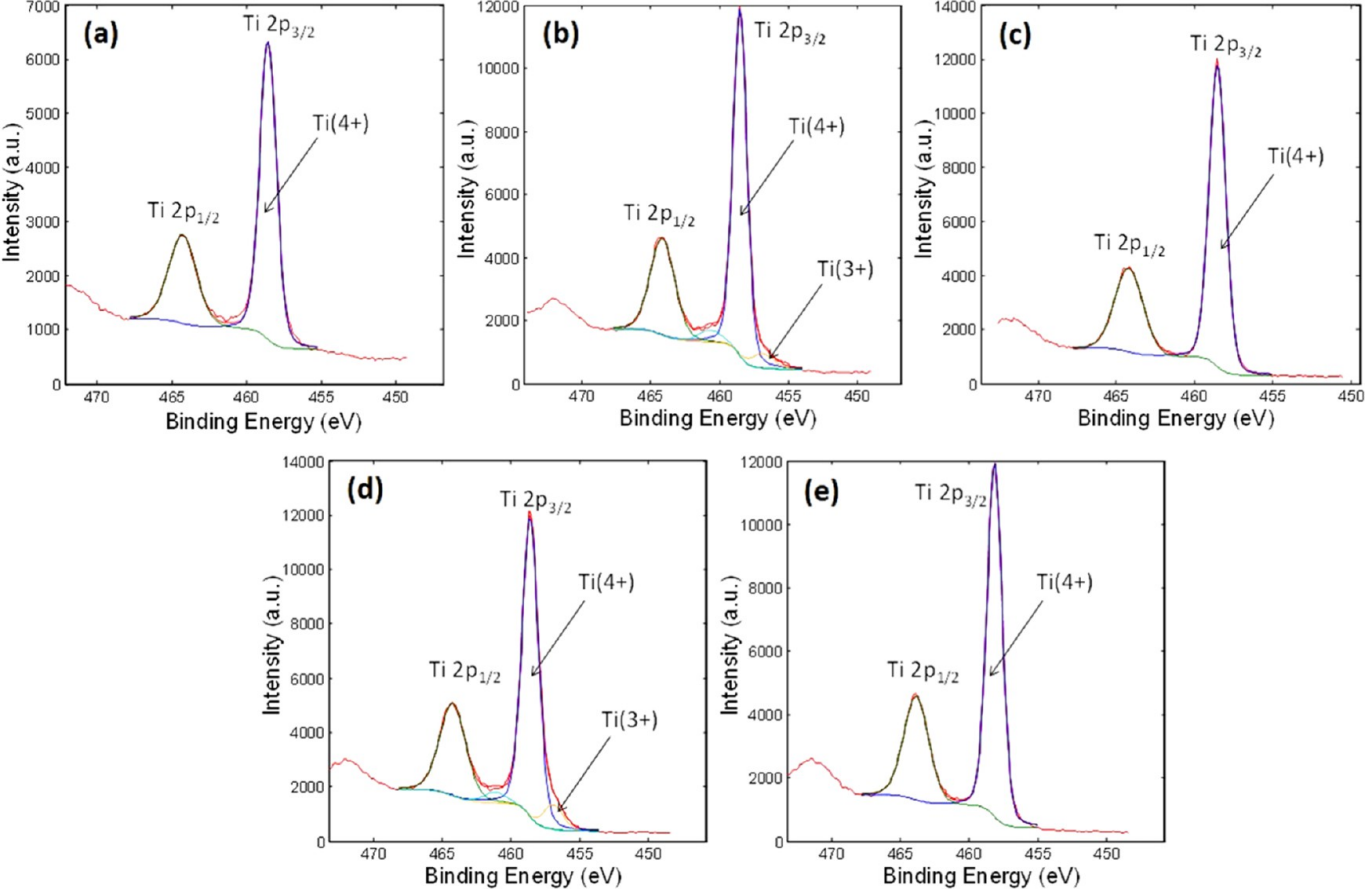
XPS Ti 2p spectra deconvoluted into Ti(4+) and Ti(3+) of (a) untreated Ti_6_Al_4_V; (b) Ti_6_Al_4_V + P; (c) HT; (d) HT + P; and (e) P + HT.

At the surface of all samples the high-energy resolution XPS spectra of Ti 2p were acquired, which show the oxidation states of Ti-atoms in the 3–5 nm thick surface layer. The deconvoluted Ti 2p spectra are shown in Figure 2. The main oxidation state of surface Ti-atoms is Ti(4+), related with a peak at 458.6 eV and originating from the TiO_2_, while the presence of Ti(3+) oxidation states (peak at 456 eV) was only found for samples treated with plasma in the final stage (Ti_6_Al_4_V + P and HT + P). The presence of Ti(3+) states is about 8–9% of the total Ti surface atoms (Table 1). The Ti(3+) oxidation states may exhibit different surface reactivities, which is reflected also in the biocompatibility of these samples. The correlation between surface wettability and different Ti ion states in TiO_2_ was studied by Kuscer et al.;^48^ it has been shown that surfaces with a higher concentration of Ti (3+) states are more hydrophilic compared to surfaces with a higher number of Ti(4+) states. In our case, this is hard to observe as only Ti + P and HT + P exhibit Ti(+3) states, and the HT sample was found to be the most hydrophilic. In addition, nanotopography is another surface feature that may influence surface wettability and is in our case present only on HT samples (HT, HT + P, and P + HT). Another relevant chemical group is OH, as increase in hydrophilicity was consistent with increase in Ti–OH on various surfaces.^49^ In addition, carbohydrate contamination will also affect the surface energy and was the highest for the untreated and plasma-treated surface.

**Figure 3:**
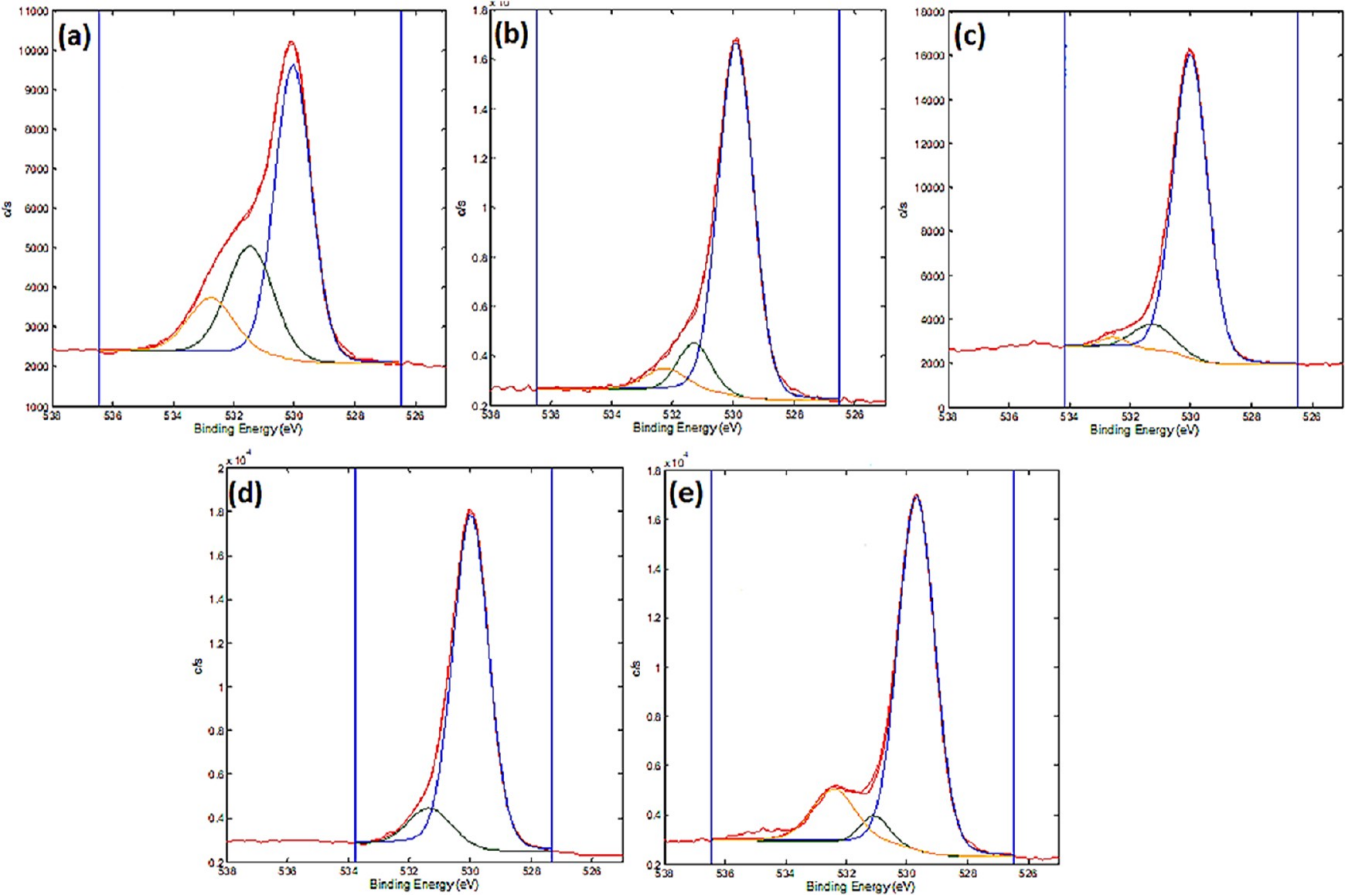
XPS O 1s spectra deconvoluted into a peak for O(2−) at 529.9 eV (blue), a peak for OH and/or oxygen vacancies at 531.2 eV (green) and a peak for adsorbed H_2_O/C–O at 532.5 eV from (a) untreated Ti_6_Al_4_V (b) Ti_6_Al_4_V + P (c) HT, (d) HT + P and (e) P + HT.

The acquired high-energy resolution XPS spectra of O 1s also indicate different states of oxygen depending on the surface treatment procedure (Figure 3). The main component at 530.0 eV belongs to the O_2_– oxygen atoms, which is correlated with Ti–O bonds. The untreated surface exhibits the largest amount of OH and/or oxygen vacancies, which are attributed to the peak at 531.2 eV, while the lowest numbers of these groups are present on the P + HT sample. A similar number of these groups is however present on P, HT, and HT + P.

On the other hand, the adsorbed H_2_O/C–O belonging to the peak at 532.5 eV seems to be the highest for the untreated Ti_6_Al_4_V and P + HT sample. However, in this case we should emphasize that the O 1s peak at 532.5 eV in case of untreated Ti_6_Al_4_V includes a much higher concentration of carbon (present as impurities), which can be seen from Table 1. The concentration of C in untreated Ti_6_Al_4_V is more than three times higher than in the case of the P + HT sample; thus, it can be stated that much more adsorbed water is present in the P + HT compared to the untreated sample. Lu et al.^49^ found that both OH and Ti–OH quantities influence the biological response for bone-forming cells (a higher concentration was shown to be favorable). Furthermore, chemical differences between the samples are evident and could be appropriately tuned to achieve the desired surface functionality (biological response, surface wettability, and aging).

**Figure 4:**
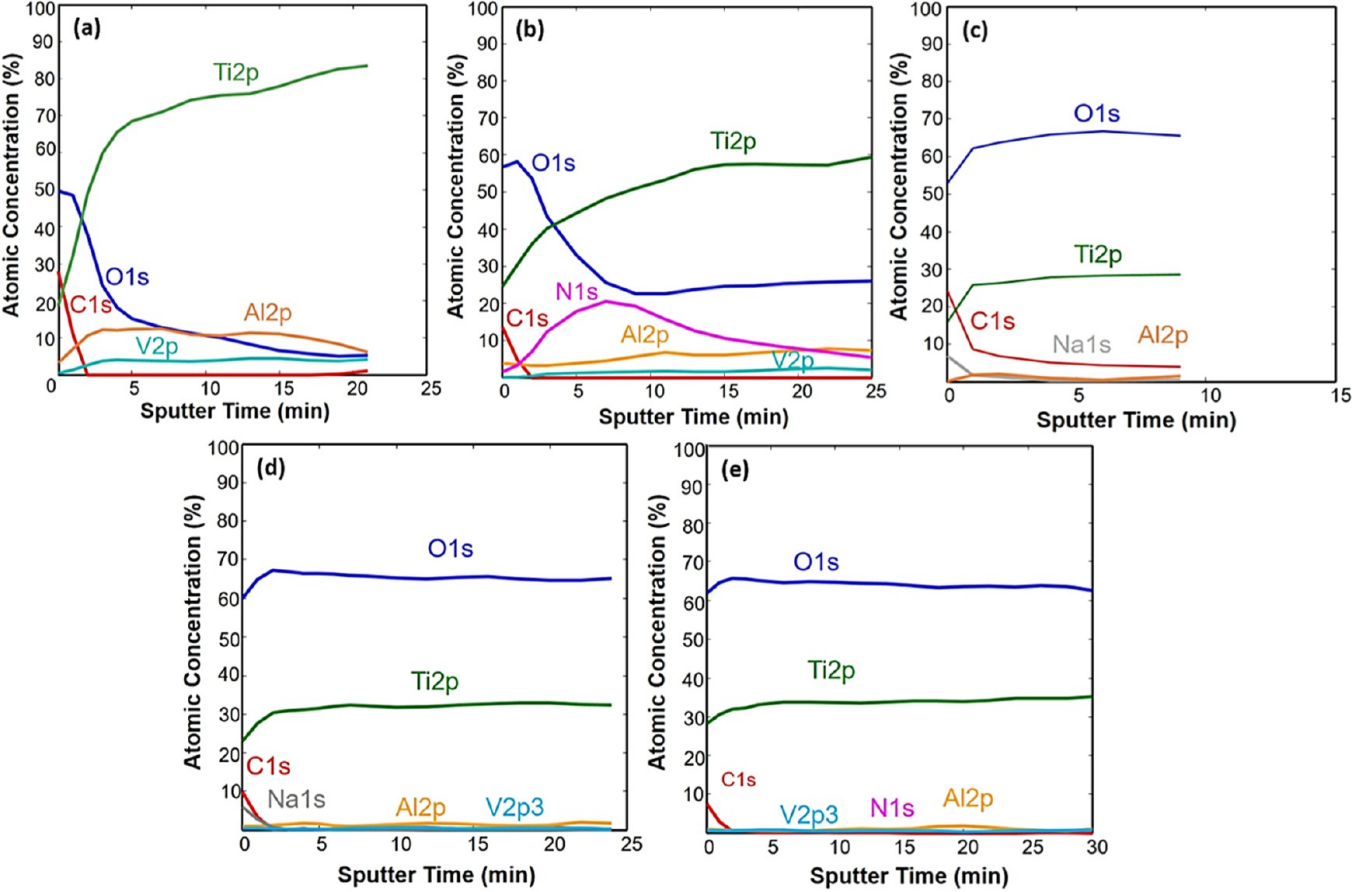
XPS depth profiles of (a) untreated Ti_6_Al_4_V, (b) Ti_6_Al_4_V + P, (c) HT, (d) HT + P, and (e) P + HT.

The XPS depth profiles of elements for the untreated Ti_6_Al_4_V, Ti_6_Al_4_V + P, HT, HT + P, and P + HT samples are presented in Figure 4. The sputtering rate was about 1 nm/min, meaning that a depth of about 20–30 nm was analyzed in this way. XPS depth profiles show that the surface is covered with oxide layer, which was TiO_2_ as evidenced from the Ti 2p spectra being at 458.6 eV (Figure 4), which is characteristic for the Ti(4+) oxidation state of Ti. On the untreated Ti_6_Al_4_V, the oxide layer was about 4 nm thick, while beneath it Al and V were present in addition to Ti (Figure 4a). The carbon on the surface is mainly due to surface contamination. After plasma treatment (Figure 4b), the oxide layer TiO_2_ increased slightly in thickness and a nitrogen-rich layer appeared beneath the surface oxide layer, which could be due to the small presence of N_2_ gas in the plasma reactor, exposure of the sample after plasma treatment to air, or even the nitrogen diffusion to the surface from the material itself (in Ti_6_Al_4_V, less than 0.05 wt % of N was present). Figure 4c–e show the XPS depth profiles of samples, which involve HT treatments. The common feature of these samples is a relatively thick oxide TiO_2_ layer. In this layer, a small concentration of Al is present, but no V was detected in the oxide. The concentration of Al is only 1–2 atomic %, which is much less than the nominal concentration of 6 atomic % of Al in Ti_6_Al_4_V. This shows that preferential formation of TiO_2_ oxide layer was obtained by HT treatment.

From XPS analysis, it was observed that after hydrothermal and/or plasma treatment, the concentrations of Ti and O increase (Table 1), while the thickness of the oxygen layer, according to XPS depth analysis, was much higher for all HT samples (Figure 4). According to EDS analysis, we could presume that the highest oxide layer was formed on the P + HT sample (Table 2). The results from Table 2 show that the amount of oxygen increases in the order HT < HT + P < P + HT, while practically no oxide was detected on the untreated Ti_6_Al_4_V and Ti_6_Al_4_V + P sample, which is in accordance with the results from the XPS depth profile (Figure 4). Interestingly, in the case of HT + P and P + HT, no V was detected, while on all HT samples practically no Al was found, but a small amount of Na, due to the use of NaOH for the HT procedure. All these may influence the platelet adhesion and activation on surfaces, as already described in some works,^50,51^ or may influence the interaction with other cell types.

**Table 2:** Surface Chemical Composition in Atomic % Obtained by EDS Analysis of Ti_6_Al_4_V Samples

	atomic %				
material	O	Na	Al	Ti	V
Ti_6_Al_4_V			9.8	87.7	2.5
Ti_6_Al_4_V + P			9.3	88.1	2.6
HT	28.2	7.7		60.6	3.5
HT + P	40.4	6.9	0.3	52.4	
P + HT	49.1	7.9		43.0	

**Figure 5:**
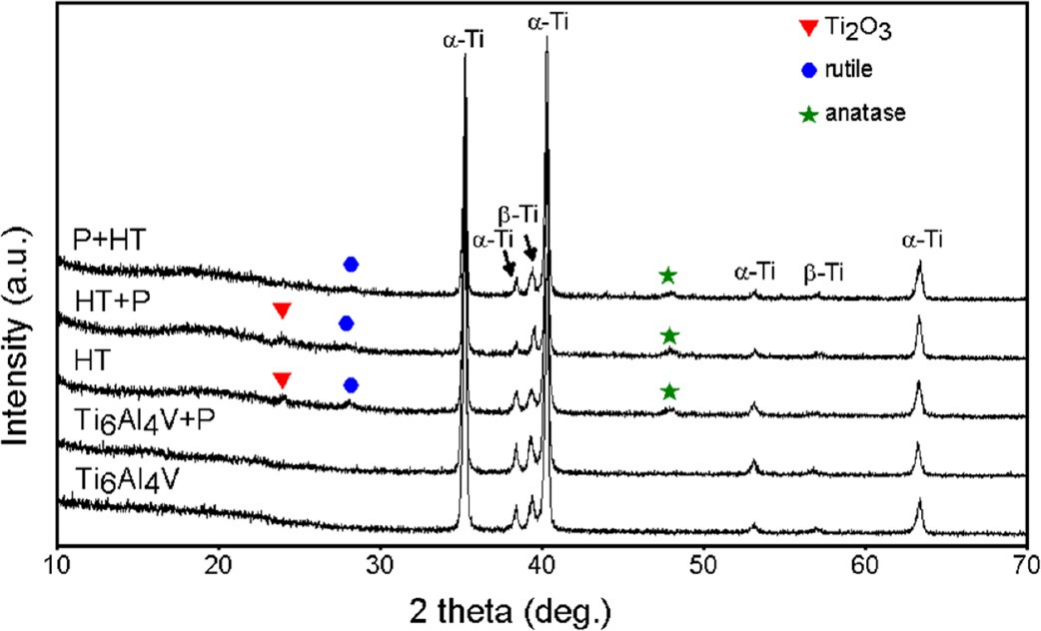
XRD spectra of Ti_6_Al_4_V samples.

From the XRD spectra presented in Figure 5, peaks of α–Ti and β-Ti, which are characteristic for Ti_6_Al_4_V, can be observed for all samples. The oxide crystalline phase was not detected on untreated Ti_6_Al_4_V and Ti_6_Al_4_V + P. On HT, HT + P, and P + HT samples, anatase and rutile phases, common for TiO_2_, were detected. In the case of the P + HT sample, a characteristic additional rutile peak is observed (Figure 5).

We believe that by oxygen plasma pretreatment, oxygen-containing functional groups are introduced onto the surface of Ti_6_Al_4_V, which influence the growth of the HT layer. We suspect that this can alter the nucleation and growth of TiO_2_ on the surface. After HT treatment, therefore, pure TiO_2_ (a mixture of anatase and rutile) can be formed on the surface of the P + HT sample, since no suboxides or impurities can be detected from XRD spectra. Presumably, this layer exhibits a thicker oxide layer, as seen from EDS (Table 2). In addition, suboxide Ti_2_O_3_ is present on the HT and HT + P sample, while this phase was not detected on the P + HT sample (Figure 5). The difference in crystallinity and surface chemistry may significantly influence the adsorption and binding of molecules as well as cell interaction.^52^

**Figure 6:**
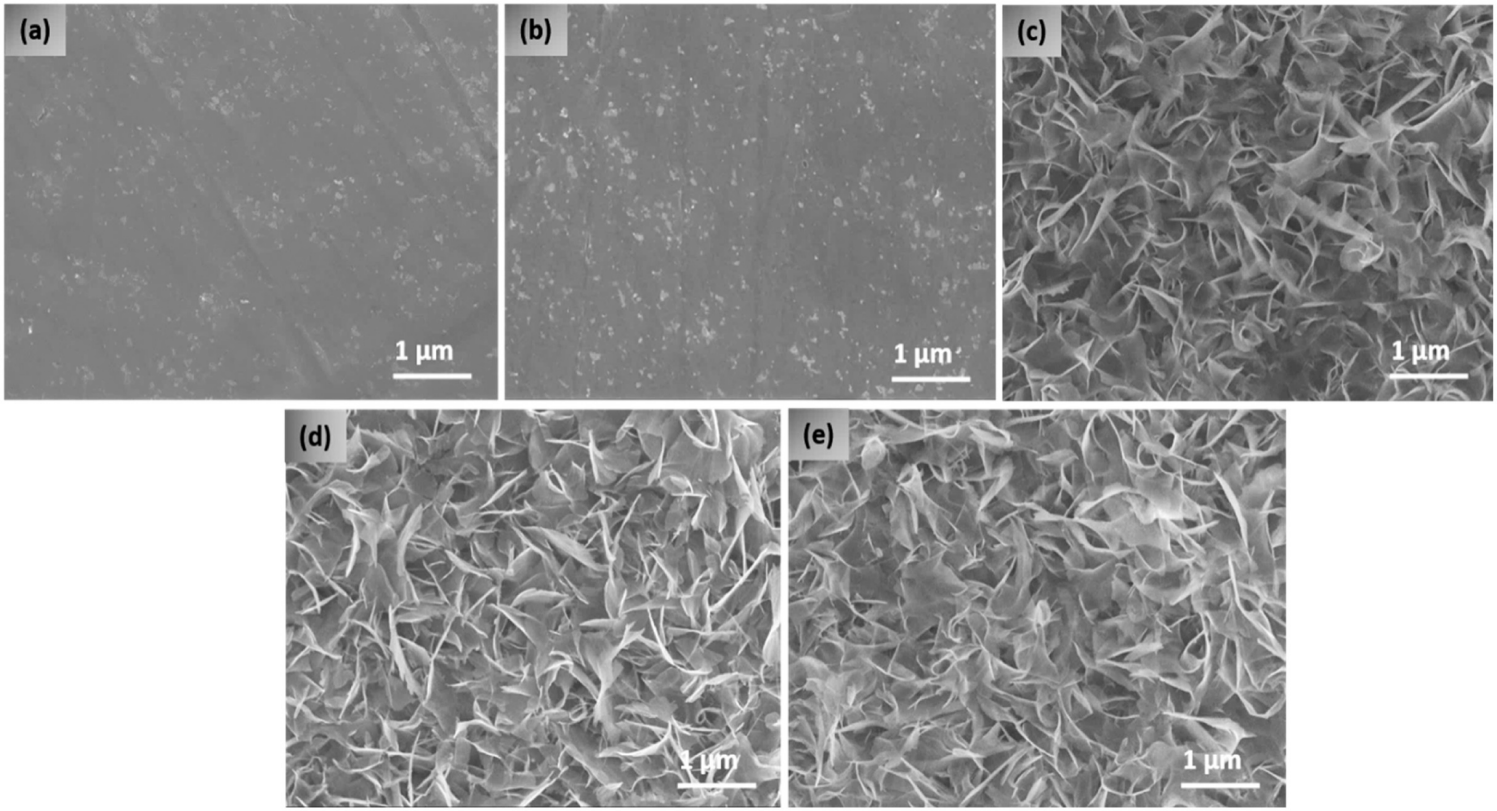
SEM images of (a) untreated Ti_6_Al_4_V, (b) Ti_6_Al_4_V treated with plasma, (c) Ti_6_Al_4_V treated with hydrothermal treatment (HT), (d) hydrothermally/plasma-treated Ti_6_Al_4_V (HT + P), and (e) plasma/hydrothermally treated Ti_6_Al_4_V (P + HT). Scale bar is 1 μm and magnification is 20,000 (inset: 10,000 magnification).

From the SEM images (Figure 6) it can be seen that practically no difference in morphology was observed between untreated Ti_6_Al_4_V and Ti_6_Al_4_V + P (Figure 6a,b). Although the HT samples also have a similar morphology, as seen in Figure 6c–e, the surface consists of feather-like micronano flake structures that are intertwined with sharp edges protruding from the surface. Hemocompatibility was studied by incubating the samples with whole blood, and subsequently, the morphology and number of platelets were inspected on the surface of untreated Ti_6_Al_4_V, Ti_6_Al_4_V + P, HT, HT + P, and P + HT. Platelet activation and adhesion can be determined by the morphological changes in addition to counting the number of cells attached on the surface. Goodman^53^ has explained that the activation of platelets on a surface can be correlated with the shape of the platelets in different stages from minimally activated to fully activated. The stages are defined as round (R), dendritic (D), spread dendritic (SD), spread (S), and fully spread (FS), of which S and FS are recognized as the activated form of platelets.

**Figure 7:**
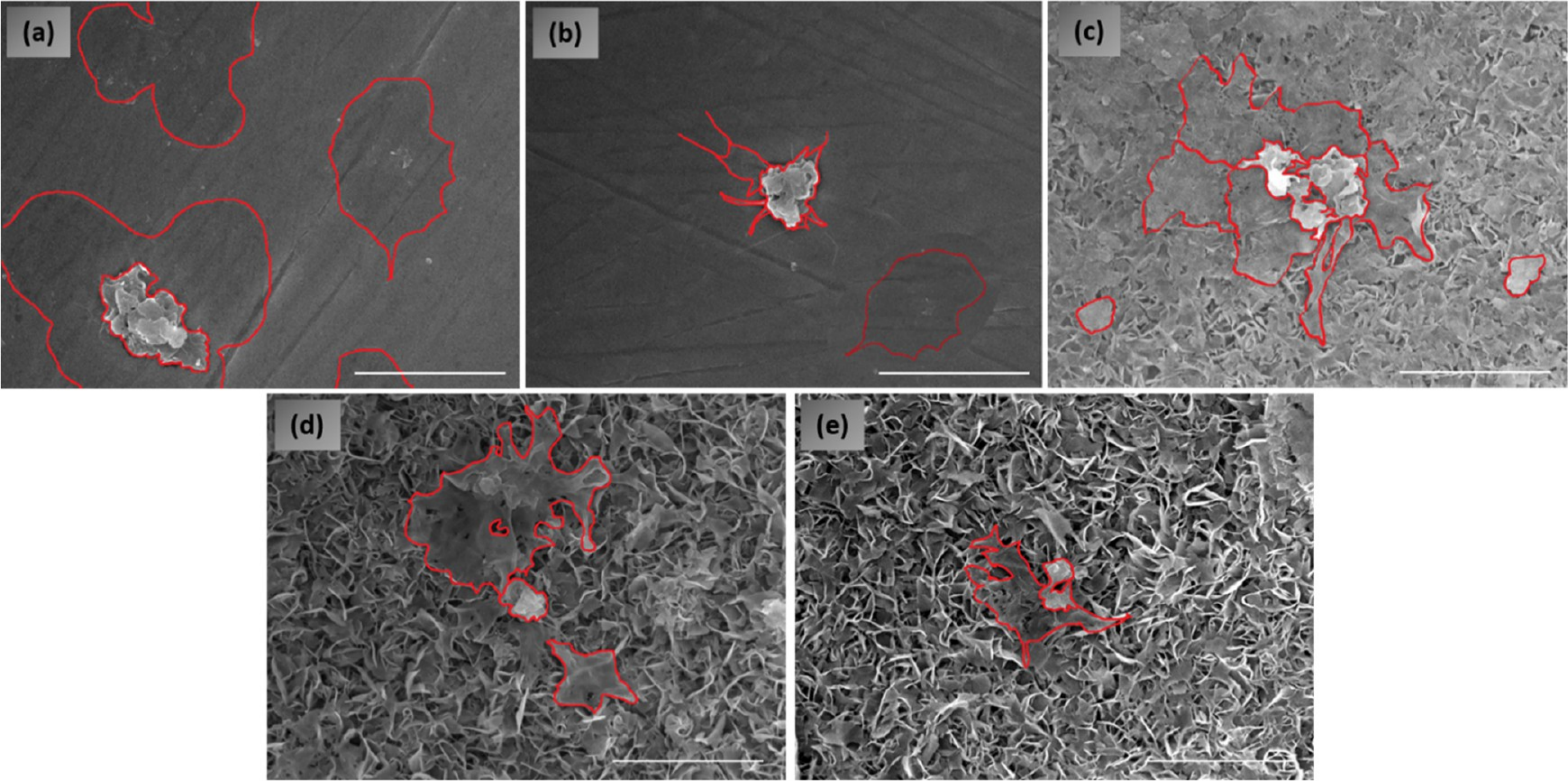
SEM images of (a) untreated Ti_6_Al_4_V, (b) Ti_6_Al_4_V + P, (c) HT, (d) HT + P, and (e) P + HT after incubation with whole blood. Scale bar is 1 μm and magnification is 5000. (Platelet shapes are marked by red color.)

Platelet interaction with sample surfaces was examined using SEM and is shown in Figure 7. Aggregates and individual platelets can be clearly observed to be distributed and fully spread on the untreated Ti_6_Al_4_V surface (Figure 7a). Upon plasma treatment (Ti_6_Al_4_V + P), a significant decrease in the number of platelets can be observed (Figure 7b). But the remaining platelets are found in both dendritic and spread forms; platelets are activated on the surface through lamellipodia and partially due to pseudopodia, indicating the fully activated form of the platelets. Analysis of platelets on HT samples was difficult, due to nanostructured surface topography. However, it could be observed that on HT samples (Figure 7c), more platelets were detected. In Figure 7c the platelets are segregated, which could with high risk lead to thrombosis. Interestingly, a lesser number of platelets was detected on HT + P and P + HT surfaces. The observed platelets were in round and dendritic form, which indicates that they are not highly activated, and they were also not segregating. It is hard to give specific conclusions of platelet interaction with these surfaces; however, it could be speculated that the HT nanostructured surface of titanium oxide fabricated by our method increases platelet interaction. It also seems that their interaction can be minimized by optimizing the top surface chemistry. It seems that platelets are sensitive to Ti(+3) states, as plasma-treated surfaces in the final stage have lower platelet adhesion compared to the control (P and HT + P).

**Figure 8:**
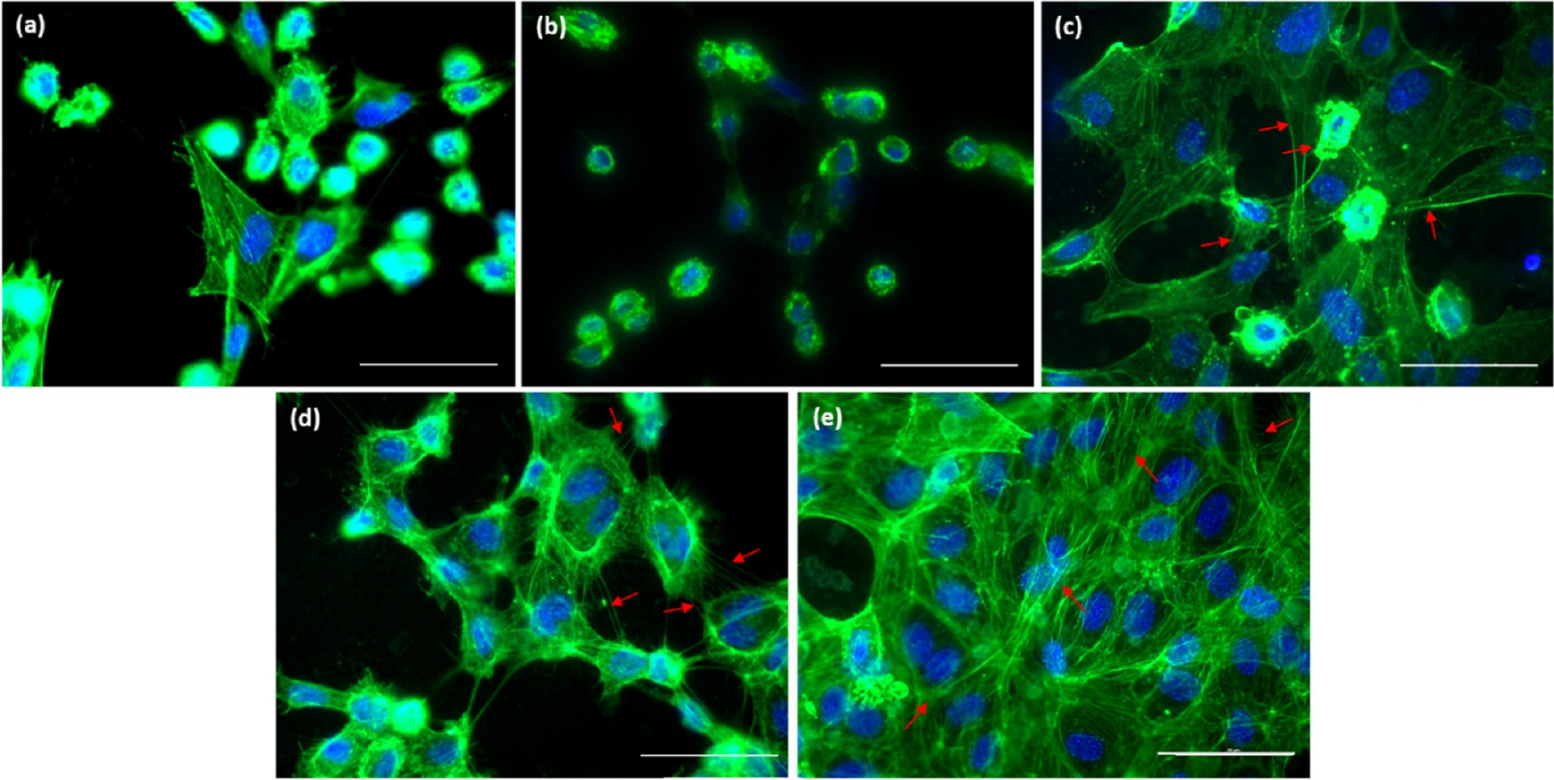
HCEC on the surface of (a) untreated Ti_6_Al_4_V, (b) Ti_6_Al_4_V + P, (c) HT, (d) HT + P, and (e) P + HT as determined by immunofluorescent microscopy. F-actin is shown in green (FITC-phalloidin). Nuclei are visualized with DAPI (blue color). Scale bar 25 μm.

The morphology of endothelial cells on the prepared samples was studied using immunofluorescent microscopy. For vascular stents, it is preferable to have good endothelialization, due to antithrombotic and antiadhesive properties; therefore, it is essential to study the growth of endothelial cells on the surfaces. The actin filaments of the HCEC (marked green, stained by phalloidin) clearly reveal the shape of cells on different surfaces in Figure 8.

**Figure 9:**
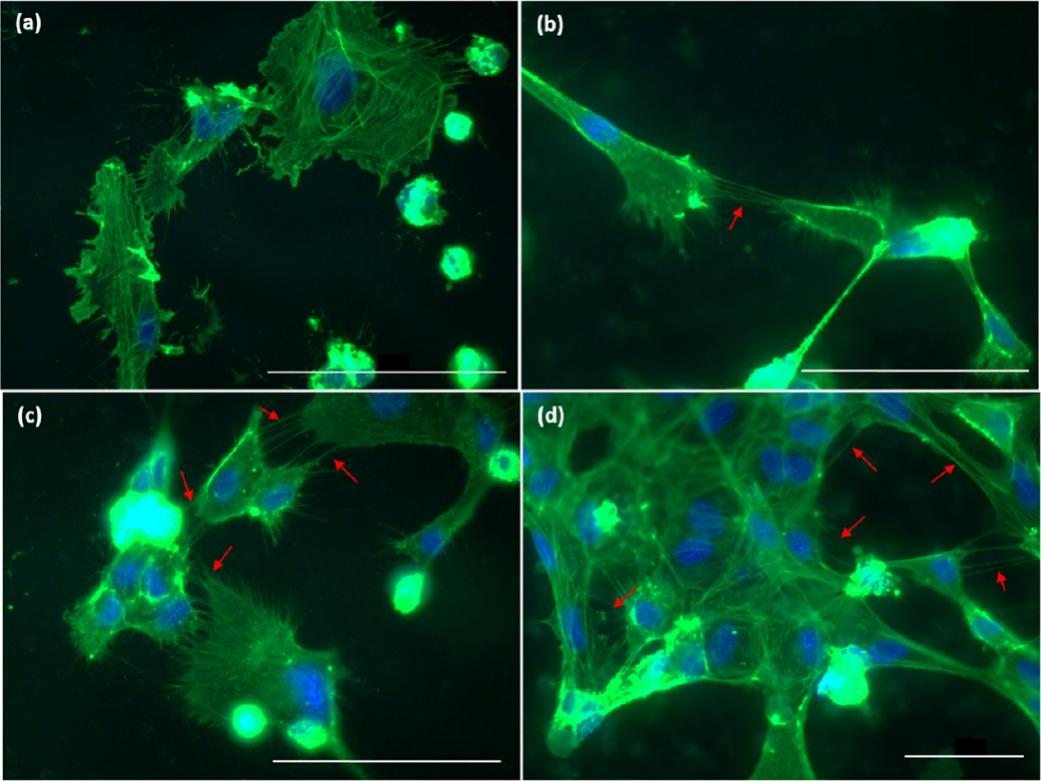
HCEC on the surface of (a) untreated Ti_6_Al_4_V, (b) HT, (c) HT + P, and (d) P + HT showing attachment through type I TNTs determined by immunofluorescent microscopy. F-actin is shown in green (fluorescein phalloidin). Nuclei are visualized with DAPI (blue color). Scale bar 50 μm.

Analysis of HCEC on the surface of untreated Ti_6_Al_4_V and Ti_6_Al_4_V + P revealed mostly a round cell shape (Figure 8a,b), although some flattened individual cells on untreated Ti6Al4 V were also observed. On the HT-treated surfaces (HT, HT + P, and P + HT) cells are flattened, and long cellular cytoplasmic extensions and cell–cell contact can be observed (Figure 8c–e), although some membrane blebbing can be seen, especially in the case of the HT + P sample. Interestingly, on the P + HT surface, cells are numerous, continuously spreading over the surface, which indicates that endothelialization is promoted on these type of surfaces. According to the results of our study, it could be concluded that HCEC cells spread well on the nanotopography created by HT treatment. However, they seem to be highly sensitive to surface chemistry, as after plasma treatment endothelial growth is visually reduced, even on surfaces that seem to have favorable nanotopographic features (HT + P). Thus, it could be suspected that they are sensitive to Ti(+3) states, which were detected only on plasma-treated surfaces (Figure 2). On the other hand, it seems that HCEAC are sensitive also to oxygen groups; the best proliferation on P + HT surfaces could be due to the high percentage of chemisorbed water on the surface of the titanium oxide layer (Figure 3), as well as the increased crystallinity and thickness of the oxide layer. Moreover, an interesting observation was found on nanostructured surfaces (HT, HT + P, P + HT), where cells seem to highly interact with each other by the so-called tunneling nanotubes (TNTs). TNTs were just recently discovered, while their complete mechanism of formation is still not fully understood. TNTs are membranous tubes that enable direct bridging of neighboring cells and may offer a very specific and effective way of intercellular communication.^54^ Two types of TNTs exist: Type I TNTs, which contain actin filaments and begin growing like filopodia, and Type II TNTs, which start to grow when two neighboring cells attached to each other start to move apart. In this case, when the tube of Type II TNT elongates, the actin gradually disappears and only cytokeratin filaments remain.^54^ In Figure 8d TNTs of Type I containing actin filaments (green) can be seen, while more detailed examples are presented in Figure 9. Interestingly, TNTs were found only in the case of nanostructured surfaces (Figure 9b–d). This actin inside the membrane nanotubes connects two neighboring cells. The reason for the high TNT formation could be the better overall grip of cells when coiling on nanostructured surfaces (like an extracellular matrix), which assist cell motility and intercellular interactions.^55^ Communication between cells is crucial for the proper functioning of multicellular organisms.^54,56^

**Figure 10:**
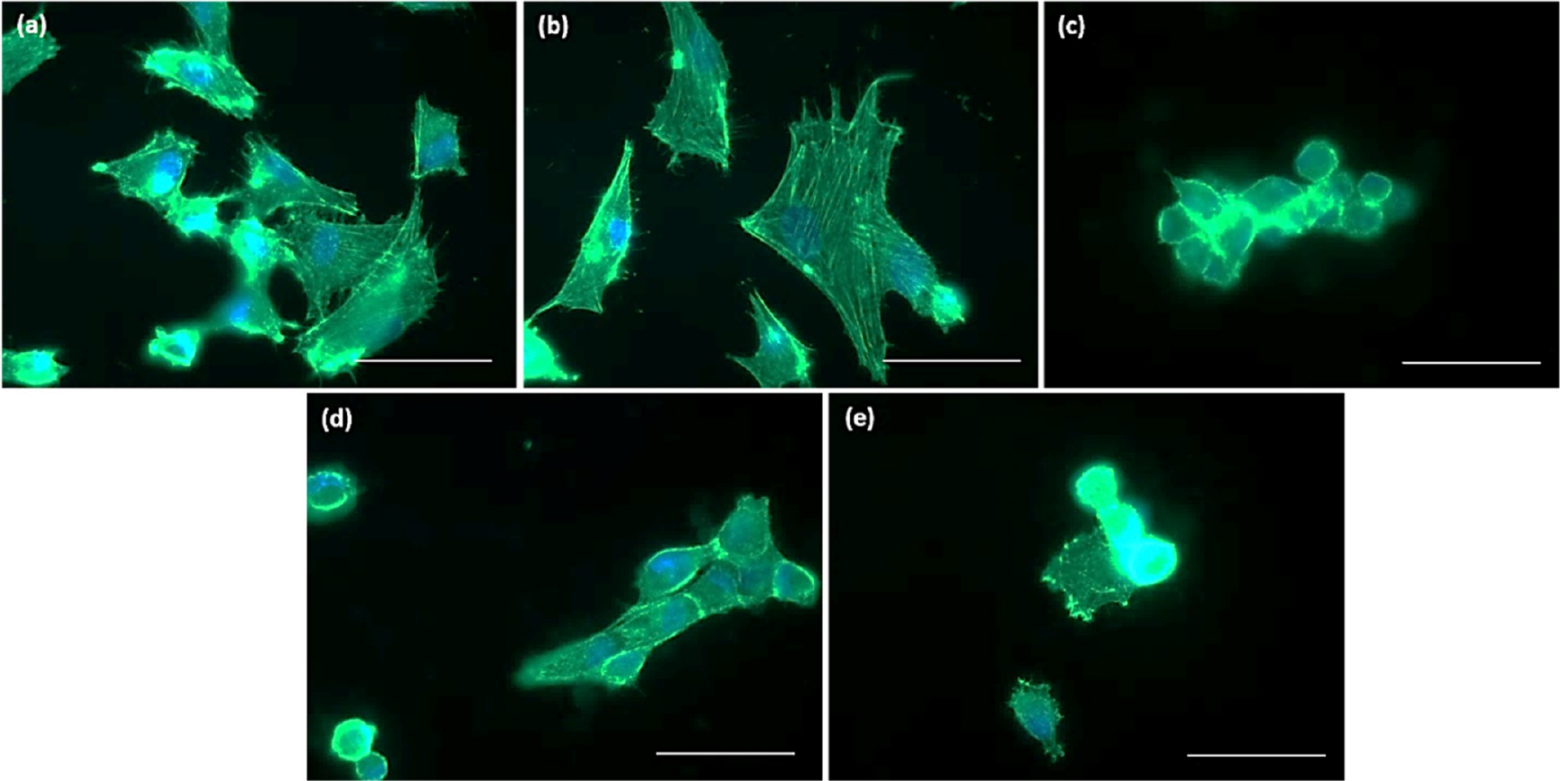
HCASMC on the surface of (a) untreated Ti_6_Al_4_V, (b) Ti_6_Al_4_V + P, (c) HT, (d) HT + P, and (e) P + HT determined by immunofluorescent microscopy. F-actin is shown in green (fluorescein phalloidin). Nuclei are visualized with DAPI (blue color). Scale bar 25 μm.

Similarly, for endothelial cells, the morphology of smooth muscle cells on the Ti_6_Al_4_V samples was studied using immunofluorescent microscopy (Figure 10). For application of vascular stents, reduced proliferation of HCASMC is desired. On the untreated Ti_6_Al_4_V and Ti_6_Al_4_V + P the cells are mostly flattened (Figure 10a,b). On the surface of HT-treated samples (HT, HT + P, and P + HT) the cells are round (Figure 10c–e), suggesting poor attachment and apoptosis. The HCASMC seems to be most noticeably affected by surface nanotopography, as their growth on all HT samples is visually decreased, which could be partially correlated also with appearance of the rutile phase and increased oxygen thickness on these surfaces. According to our study, the OH groups and Ti(+3) or O(−2) states seem not to play a prevailing role in HCASMC proliferation.

To further confirm the changes in cell-surface interaction, the number of cells in round and spread form was evaluated from the images taken by immunofluorescent microscopy. Cells were counted on four powerfields from each slide (2 biological replicates, each producing 2 slides). The results are presented in Table 3. It can be easily noticed that a high number of round HCEC cells (which presumably are mostly apoptotic, as it would be unusual that all would be dividing at the same time) was found on the surface of Ti_6_Al_4_V and Ti_6_Al_4_V + P, while a much higher number of spread cells was detected on nanostructured surfaces (HT, HT + P, P + HT). The highest number of spread cells was observed for the P + HT surface. In the case of HCASMC, the opposite observation was found. The highest number of round cells was found on nanostructured surfaces, and the lowest on Ti_6_Al_4_V and Ti_6_Al_4_V + P.

**Table 3:** Number of Round and Spread HCAEC and HCASMC in Different Samples

	endothelial cell		smooth muscle cell	
sample	round	spread	round	spread
Ti_6_Al_4_V	13	2	3	12
Ti_6_Al_4_V + P	26	4	1	6
HT	4	10	38	1
HT + P	5	15	10	4
P + HT	4	19	48	1

Legend: median number of cells (min-max) is presented from 4 slides/2 biological replicates.

## 4 Conclusions

Interaction of surfaces with biological materials (platelets, endothelial, and smooth muscle cells) was shown to be highly influenced not only by the surface nanotopography but also by the specific surface chemistry. Platelet interaction seems to be reduced on plasma-treated surfaces, where an increased concentration of Ti (+3) states was observed. To sum up, the least-activated platelets were observed on the surface of plasma-treated Ti_6_Al_4_V + P and HT + P, while nanostructuring alone (by HT treatment) seems not to reduce platelet activation. Thus, the results of the platelet interactions with the samples prepared in this study indicate that plasma treatment could have beneficial effects on platelet adhesion. However, it should be noted that platelet adhesion was not studied on aged samples, which might also influence platelet-surface interaction, as the wettability of plasma-treated surfaces was shown to be rapidly altered over time. Moreover, adhesion and proliferation of endothelial and smooth muscle cells on Ti_6_Al_4_V samples appear to be surface morphology dependent. Endothelial cells readily adhere and proliferate on nanostructured surfaces (HT, HT + P, and P + HT). The opposite was observed for smooth muscle cells, which lost their spindle shape and became round (on nanostructured surfaces), indicating apoptosis. Our study indicates that endothelial cells are highly sensitive to surface chemistry, the opposite to platelets; they seem not to attach and proliferate well on surfaces containing Ti(+3) states. In addition, the increased proliferation on the P + HT sample could be correlated also with the thicker titanium oxide layer, high rutile content, and increased concentration of chemisorbed water. This study provides relevant data for future developments of a multifunctional vascular stent surface; by fine-tuning the surface parameters, growth of endothelial cells could be promoted, while at the same time reducing the proliferation of smooth muscle cells and adhesion and activation of platelets. Furthermore, studies conducted on cellular differentiation could profit from this work.
